# Laparoscopic Cholecystectomy for Gall Bladder Volvulus: A Report of an Original Case With Review of Literature

**DOI:** 10.1155/2024/9319605

**Published:** 2024-11-04

**Authors:** Ayad Ahmad Mohammed

**Affiliations:** Department of Surgery, College of Medicine, University of Duhok, Duhok, Kurdistan Region, Iraq

**Keywords:** acute abdomen, gall bladder volvulus, laparoscopic cholecystectomy, torsion of gall bladder

## Abstract

**Background:** Volvulus of gallbladder is defined as a rotation of the gallbladder on its mesentery along the axis of the cystic duct and cystic artery. Many factors are postulated to be the causes such as anatomical, mechanical, physiological, and hormonal risk factors but the presence of a distended gallbladder with a redundant mesentery is thought to be an important cause.

**Case presentation:** A 68-year-old woman presented with right hypochondrial pain and vomiting for 2 days that was radiated to the interscapular region and associated with nausea and vomiting. The patient had no jaundice and the abdominal examination showed severe tenderness with guarding during palpation of the right upper abdomen with no palpable mass. The WBCs were elevated, with normal liver enzymes, bilirubin, and alkaline phosphatase. The ultrasound showed a single gallstone with increased wall thickness. There was no significant clinical improvement with antibiotics and analgesics. During laparoscopy, volvulus of the gallbladder was discovered causing gangrene of the gallbladder. Laparoscopic detorsion and successful laparoscopic cholecystectomy were performed. The patient was discharged on the third postoperative day with dramatic improvement with no postoperative complications.

**Conclusion:** Gall bladder volvulus is an acute surgical emergency that is usually seen in the elderly population. It required a high index of suspicion especially in the absence of gallstones and must be differentiated from acalculous cholecystitis. Most cases are discovered at surgery. It must be managed with immediate detorsion and cholecystectomy, and the prognosis is excellent in most cases after an appropriate surgical intervention.

## 1. Introduction

Volvulus of the gallbladder is the rotation of the gallbladder on its mesentery along the axis of the cystic duct and cystic artery. The presence of distended gallbladder with a redundant mesentery is thought to be an important cause. Sometimes the presence of a long mesentery with a narrow attachment to the liver may result in floating gallbladder which is liable for rotation and torsion [1–3].

More than 85% of the cases occur in patients between 60 and 80 years of age, there is female predominance, and the female-to-male ratio is reported to be 3:1. Patients usually present with acute right upper quadrant abdominal pain that mimics acute cholecystitis, nausea, vomiting, and fever are common symptoms. The inflammatory markers are usually elevated [4, 5].

CT scan of the abdomen may reveal a distended and horizontally displaced gallbladder with a thickened, hyperattenuating, and poorly enhanced walls. A “whirl sign” of the cystic pedicle is pathognomonic for a gallbladder volvulus. Magnetic resonance imaging (MRI) and magnetic resonance cholangiopancreatography (MRCP) may demonstrate necrosis in the wall of the gallbladder or infarction. Hepatobiliary iminodiacetic acid (HIDA) scan findings resemble a bull's-eye because of the accumulation of radioactive tracer within the gallbladder [6].

Gall bladder volvulus is an acute surgical emergency, most cases are discovered during surgery, and a high index of suspicion is required for the diagnosis. The rate of preoperative diagnosis may reach 30%. It must be managed with immediate detorsion and cholecystectomy, and the prognosis is excellent in most cases after an appropriate surgical intervention. There are reported cases in literature, and most are diagnosed during surgery [2, 4, 7–9].

## 2. Case Presentation

A 68-year-old woman presented to the emergency department complaining of right hypochondrial pain and vomiting for 2 days. The pain was colicky in nature at the start and then became continuous pain that was felt at the right hypochondrial region and radiated to the interscapular region and was associated with nausea and vomiting. The fever was intermittent.

The patient was admitted to the hospital and received intravenous antibiotics and analgesics.

General examinations showed high blood pressure (150/90 mm·Hg), a normal pulse rate was 90 beats/minute, and the temperature was 37.6 degree Celsius. The patient was not jaundiced with no pallor. Abdominal examination showed severe tenderness with guarding during palpation of the right upper abdomen. No organomegaly was detected with no masses.

The investigations showed elevated white blood cell levels of 13,400 cells/L, and liver enzymes showed normal SGPT (23 IU'L), SGOT (31 U/L), and alkaline phosphatase (120 U/L). The total bilirubin level was normal (0.73 mg/dL).

**Figure 1:**
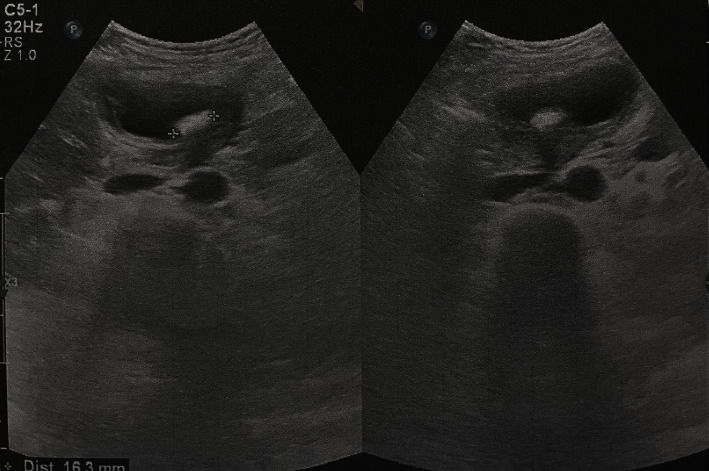
Ultrasound picture of the gallbladder showed an increased wall thickness and a single stone inside the cavity of the gallbladder.

The ultrasound showed a single stone 16 mm in diameter in the cavity of gallbladder with increased wall thickness (7 mm). Other intra-abdominal organs were normal as shown in Figure 1.

**Figure 2:**
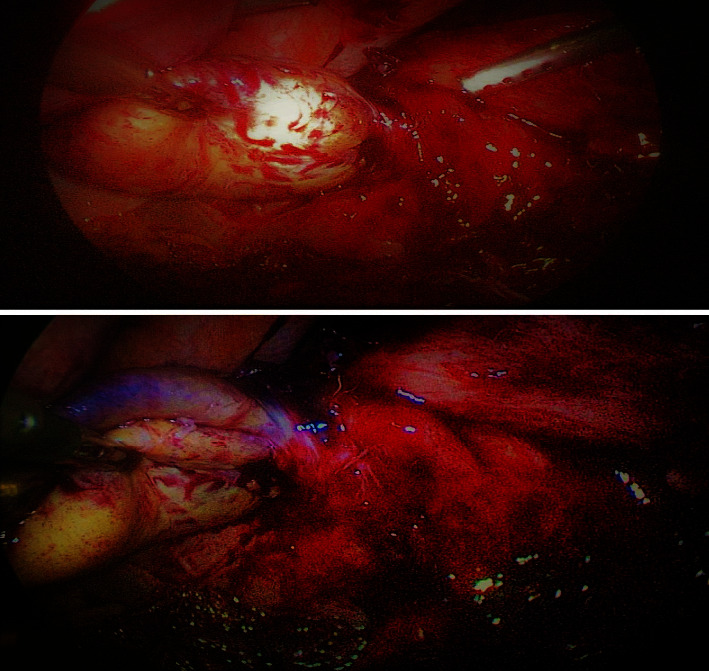
Volvulus of the gallbladder with gangrene of the gallbladder.

There was no significant clinical improvement on conservative therapy. At the fifth day, the decision was done for surgery. Laparoscopy was performed, and during surgery, there were adhesions between the gallbladder and the omentum, adhesions were released, and volvulus of the gallbladder was found causing gangrene of the gallbladder as shown in Figure 2.

Laparoscopic detorsion of the gallbladder was done, and successful laparoscopic cholecystectomy was performed. Tube drain was placed in the subhepatic region due to extensive dissection and gangrene of the gallbladder, so drain was placed to drain out any fluid after surgery.

The patient was discharged on oral antibiotics on the third postoperative day with dramatic improvement with no postoperative complications.

## 3. Discussion

Gall bladder volvulus is very rare, and the exact incidence is underestimated because it is underreported, and all the present information is from case reports. It is mostly seen in elderly population, although the case is reported in younger age population, with smallest reported age around 2 years. This condition is very rare and is described for the first time by Wendel in 1898 in a 25-year-old pregnant lady, and exact prevalence is not reported; however, less than 400 cases are reported in literature worldwide [1–3, 7].

Four types of anatomic variations of gallbladder have been reported that might predisposed to volvulus, the first one is a failure of the pars cystica to migrate normally from the hepatic diverticulum during week 4 to week seven of embryological life resulting in a complete absence of gallbladder mesentery and free-floating gallbladder that is suspended by the cystic duct and artery alone, the second anomaly is long mesentery of gallbladder as a consequence of aging process making the gallbladder mobile and thus increasing the risk of its torsion especially when there is atrophy of the liver, in the third anomaly, the fundus of the gallbladder is detached from the liver bed leading to increase its mobility and higher risk of torsion, and the fourth and the rarest one is that in which a normal fixed gallbladder is attached to a mobile hepatic lobe [7].

Volvulus might be complete (180° torsion or less) or incomplete (greater than 180°), and it might be clockwise or anticlockwise organoaxial torsion, and it occurs along the longitudinal axis of the gallbladder involving both the cystic artery and the cystic duct. The majority of cases have no associated gallstones, and this makes a difficult to differentiate this condition from acalculous cholecystitis [4, 7].

The predisposing factors include advanced age, female sex, weight loss, atrophy of the liver, kyphoscoliosis, and loss of visceral fat which results in the elongated gallbladder mesentery and loss the fatty support for the gallbladder resulting in torsion. Some mechanical events may also facilitate gallbladder volvulus like sudden shifts in body position or intense gallbladder contraction due to heavy fatty meals [4].

It is usually diagnosed during surgery in most of the cases; however, it may be possible to diagnose the condition preoperatively with proper imaging like ultrasound or CT scan. Doppler ultrasound, although not routinely done, shows the absence of blood flow in the cystic artery [4, 6].

The condition should be suspected in the elderly, thin females with spinal deformities, who presented with acute right upper quadrant abdominal pain with or without vomiting, and with palpable abdominal mass with no toxemia. Patients may have repeated previous attacks of right hypochondrial pain that resolved spontaneously [4, 7].

The prognosis is excellent after appropriate intervention. The mortality rate is reported to be less than 5% and is dependent on the associated comorbidities. If there is delay in the diagnosis and management, it may result in gangrene and perforation of the gallbladder and sepsis. More case studies should be done to define the presentation for better preoperative diagnosis [3, 10].

## Data Availability

Data will be available on request.
